# Echinocandins: A ray of hope in antifungal drug therapy

**DOI:** 10.4103/0253-7613.62396

**Published:** 2010-02

**Authors:** Neeta D. Grover

**Affiliations:** Department of Pharmacology, Bharati Vidyapeth University Medical College and Hospital, Sangli, Maharashtra, India

**Keywords:** Anidulafungin, candidemia, caspofungin, echinocandins, esophageal candidiasis, invasive aspergillosis, invasive candidiasis, micafungin

## Abstract

Invasive fungal infections are on the rise. Amphotericin B and azole antifungals have been the mainstay of antifungal therapy so far. The high incidence of infusion related toxicity and nephrotoxicity with amphotericin B and the emergence of fluconazole resistant strains of *Candida glabrata* egged on the search for alternatives. Echinocandins are a new class of antifungal drugs that act by inhibition of β (1, 3)-D- glucan synthase, a key enzyme necessary for integrity of the fungal cell wall.

Caspofungin was the first drug in this class to be approved. It is indicated for esophageal candidiasis, candidemia, invasive candidiasis, empirical therapy in febrile neutropenia and invasive aspergillosis. Response rates are comparable to those of amphotericin B and fluconazole. Micafungin is presently approved for esophageal candidiasis, for prophylaxis of *candida* infections in patients undergoing hematopoietic stem cell transplant (HSCT) and in disseminated candidiasis and candidemia. The currently approved indications for anidulafungin are esophageal candidiasis, candidemia and invasive candidiasis.

The incidence of infusion related adverse effects and nephrotoxicity is much lower than with amphotericin B. The main adverse effect is hepatotoxicity and derangement of serum transaminases. Liver function may need to be monitored. They are, however, safer in renal impairment. Even though a better pharmacoeconomical choice than amphotericin B, the higher cost of these drugs in comparison to azole antifungals is likely to limit their use to azole resistant cases of candidial infections and as salvage therapy in invasive aspergillosis rather than as first line drugs.

## Introduction

Candidemia is a major contributor to mortality in immunosuppressed patients (33 to 47%), due to a rise in invasive fungal infections.[1,2] Although *C. albicans* is the most commonly found isolate, patients infected with *C. albicans*-show the highest mortality.[3] They also show a reduced susceptibility to azole antifungals.[2,4] Fluconazole, which has been the mainstay of treatment till now, is losing ground due to increasing resistance by *C.glabrata*.[5]

Aspergillosis is a major cause of morbidity in patients with hematological malignancies and those undergoing transplants.[6] Amphotericin B, which is used against invasive fungal infections, has a very high frequency of infusion related adverse effects and nephrotoxicity. Voriconazole is the only azole antifungal drug licensed for use in invasive aspergillosis. The introduction of echinocandins, a new class of antifungals, against this backdrop, is a promising development in antifungal therapy.

Echinocandins are a group of semisynthetic, cyclic lipopeptides with an N-linked acyl lipid side chain. The drugs in the class are: caspofungin, micafungin and anidulafungin.

## Mechanism of action

The echinocandins act as non competitive inhibitors of β - (1, 3) - D-glucan synthase, an essential component of the fungal cell wall that is not present in mammals. Inability of the organism to synthesize β - (1, 3) - D-glucan leads to osmotic instability and cell death.[7–10]

## Antifungal spectrum

Echinocandins exhibit good fungicidal activities against *Candida albicans, Candida parapsilosis* and *Candida guilliermondii*. Good activity is also shown against amphotericin B-resistant and fluconazole-resistant *Candida glabrata*. For Aspergillus species, echinocandins have fungistatic activity in contrast to amphotericin B and trizoles, which exhibit fungicidal activity.[11–13] Echinocandin resistance, though not common, has been reported in *C. glabrata* and *C. parapsilosis*.[14,15]

## Pharmacokinetics

All echinocandins have poor oral bioavailability and are administered only by the intravenous route. Since none of the drugs are excreted solely by the kidneys, the dose need not be altered in renal impairment. In moderate hepatic impairment, the dose of caspofungin needs to be altered but this adjustment is not needed for anidulafungin and micafungin.[16–18]

### Clinical studies

Clinical studies have been conducted with all three echinocandins in various fungal infections. Caspofungin was the first drug in the group to be tested clinically. Subsequently, micafungin and anidulafungin have also been studied extensively. The various indications in which echinocandins have been studied are described below. The Food and Drug Administration (FDA)-approved indications and doses are presented in Table 1.

**Table 1:** US FDA-approved indications and dosages of echinocandins

*Drug*	*Indication*	*Dose*	*Duration*
Caspofungin[16]	Esophageal candidiasis	50 mg IV daily	Mean duration in trials 9 days. Range = 7-21 days
	Candidemia and invasive candidiasis	50 mg IV daily	Continued till 14 days after last positive culture
	Febrile neutropenia	70mg IV loading dose on day 1, followed by 50 mg IV daily	Continued till resolution of neutropenia. If fungal infection occurs, then minimum 14 days. To be continued for at least 7 days after symptoms resolve.
	Invasive Aspergillosis	70mg IV loading dose on day 1, followed by 50 mg IV daily	Based on severity of the underlying disease.
Micafungin[17]	Esophageal candidiasis	150 mg IV daily	Mean duration in patients treated successfully = 15 days. Range = 10-30 days
	Prophylaxis of HSCT patients	50 mg IV daily	Mean duration in patients treated successfully = 19 days. Range = 6-51 days
	Candidemia, disseminated candidiasis, candida peritonitis and abscess	100 mg IV daily	Mean duration in patients treated successfully = 15 days. Range = 10-47 days
Anidulafungin[18]	Esophageal candidiasis	100 mg IV loading dose on day 1, followed by 50 mg IV daily	Minimum 14 days and for at least 7 days following resolution of symptoms
	Candidemia and invasive candidiasis	200 mg IV loading dose on day 1, followed by 100 mg IV daily	14 days after last positive culture

### Adverse effects

Infusion related reactions (facial flushing, swelling, rash, pruritis, and fever) have been reported with all the echinocandins.[16–19] They usually occur immediately after infusion and respond well to antihistamines. The drug need not be withdrawn but the rate of infusion should be decreased. Thrombophlebits may occur with all the three drugs. Overall, the infusion-related events seem to be much fewer than those due to amphotericin B.[20,21] Derangement of hepatic transaminases has been observed in almost all the studies of adverse events of the echinocandins.[16–20] Liver function should be monitored, especially in case of caspofungin. However, the gross disturbance in creatinine clearance, as observed with amphotericin B, is not seen and these drugs are probably safer than amphotericin B in case of renal impairment.[16–18] Overall, the tolerability profile of echinocandins seems to be comparable to that of fluconazole[22–25] and better than that of amphotericin B.[21,26,27]

## Use in special populations

No dose adjustment is required for renal impairment including in patients on dialysis since echinocandins are highly protein bound and non dialyzable.[16–18] Patients with mild hepatic insufficiency do not require dose adjustment for caspofungin but reduction of dose to 35 mg is recommended in moderate hepatic insufficiency.[16] However, if clinical situation warrants, a 70 mg loading dose should still be administered on day one. No dose adjustment is needed for micafungin and anidulafungin in mild to moderate hepatic insufficiency.[17,18] Patients developing abnormal liver function tests while on micafungin should be evaluated for the risk/benefit ratio of continued treatment with micafungin.[17] Anidulafungin is not metabolized by the liver and no increase in its serum levels has been observed with any level of hepatic impairment.[18]

Even though echinocandins have been studied in children, none of the three drugs has yet been approved for use in pediatric population.[28–31] No dose adjustment is required for geriatric population. All echinocandins have embryo toxic potential and should be used in pregnancy only if the potential benefit to the mother outweighs the potential risk to the fetus.

## Pharmacoeconomic evaluation

A cost effectiveness analysis of caspofungin versus liposomal amphotericin B has been carried out by Bruynesteyn *et al.*[32] Cost per day for caspofungin was estimated at £ 417 for the first day (70 mg) and £ 328 per day (50 mg) from the second day onwards. The cost for amphotericin B for a 77 Kg patient (3 mg / Kg / day) was £ 483 per day. Included in the overall analysis were toxicity related costs, length of stay and quality adjusted life years (QALY). It was found that patients of suspected fungal infections treated with Caspofungin had a lower mortality in comparison with liposomal amphotericin B. Treatment with caspofungin was predicted to save 0.55 additional life years per patient. The authors concluded that caspofungin was more economical than liposomal amphotericin B in terms of cost saving as well as QALY gains.

An economic analysis of micafungin versus liposomal amphotericin B, for the treatment of candidemia and invasive candidiasis, revealed that micafungin may be a more cost effective therapy in the treatment of these conditions.[33]

It seems that using echinocandins instead of liposomal amphotericin B may be more economical, especially in patients with an impaired renal profile. But echinocandins may not be preferred over the azole antifungals, especially voriconazole.[34] For azole resistant cases; they may be more economical than liposomal amphotericin B. Even in IA, voriconazole has been found to be more cost - effective than caspofungin.[34]

## Conclusion

Keeping in mind the high cost of echinocandins in comparison to azole antifungals like fluconazole, these drugs are likely to remain as reserve drugs for most invasive candidial infections. They may be more useful in azole - resistant cases or in invasive aspergillosis as salvage therapy.
